# Reinfection and re-revision rates of periprosthetic knee infection under four different surgical strategies: a single centre retrospective observational study

**DOI:** 10.3389/fcimb.2026.1769101

**Published:** 2026-05-19

**Authors:** Changzhi Huang, Liang Lin, Xiaofeng Liu, Hong Chen, Lan Lin, Huangfeng Lin, Zida Huang, Wenming Zhang, Xinyu Fang

**Affiliations:** 1 Department of Orthopaedics Surgery, The First Affiliated Hospital of Fujian Medical University, Fuzhou, Fujian, China; 2 Department of Joint Surgery and Sports Medicine, Ningde Municipal Hospital, Ningde, Fujian, China; 3 Ningde Clinical Medical College of Fujian Medical University, Ningde, Fujian, China; 4 Department of Orthopaedics, The First Hospital of Putian City, Putian, Fujian, China; 5 The School of Clinical Medicine, Fujian Medical University, Fuzhou, Fujian, China; 6 Department of Orthopaedics, Shanghai Sixth People’s Hospital Fujian, Quanzhou, Fujian, China; 7 Clinical Research Centre for Orthopaedics Trauma and Reconstruction of Fujian Province, Quanzhou, Fujian, China; 8 Department of Orthopaedics Surgery, National Regional Medical Centre, Binhai Campus of the First Affiliated Hospital of Fujian Medical University, Fuzhou, Fujian, China; 9 Fujian Provincial Institute of Orthopaedics, the First Affiliated Hospital of Fujian Medical University, Fuzhou, Fujian, China

**Keywords:** periprosthetic joint infection, PJI, reinfection, revision, total knee arthroplasty

## Abstract

**Background:**

Management strategies for (suspected) periprosthetic joint infection after total knee arthroplasty (TKA-PJI) include debridement, antibiotics, and implant retention (DAIR); one-stage revision; 1.5-stage revision; and two-stage revision. Reported outcomes for these four approaches vary widely across studies, with failure rates ranging from 0% to 40%. This study aims to compare the four strategies in terms of reinfection rates, microbial profiles of recurrent infections, short- and long-term infection-free prosthesis survival, re-revision rates for any cause, and long-term implant loosening-free survival.

**Methods:**

We conducted a retrospective cohort analysis of 145 patients who underwent DAIR, one-stage revision, 1.5-stage revision, or two-stage revision for TKA-PJI at our centre between October 2012 and October 2022. All patients had a minimum follow-up of 2 years. Diagnosis of PJI was based on the Musculoskeletal Infection Society (MSIS) criteria for PJI.

**Results:**

After a median follow-up of 99 (35, 164)months, infection recurred in 25 cases (18.8%). Of these, 7 cases (5.3%) were defined as relapses (same organism as the initial revision), 14 cases (10.5%) as new infections (different organism), and 4 cases (3.0%) had indeterminate infection type. In 5 patients DAIR successfully eradicated the reinfection. After overall follow-up 25 patients (18.8%) underwent re-revision surgery, 17 patients (12.8%) due to an infection and 8 patients (6.0%) for aseptic reasons. The cumulative reinfection rates at 2 years after DAIR, one-stage revision,1.5-stage revision, and two-stage revision was 14.6% (95% CI: 7.8% to 15.1%), 16.0% (95% CI: 9.7% to 21.2%), 13.8% (95% CI: 8.4% to 18.9%), and 4.0% (95% CI: 3.0% to 11.1%), respectively; at 5 years was 18.4% (95% CI: 9.2% to 16.4%), 20.8% (95% CI: 11.6% to 22.2%), 17.9% (95% CI: 10.0% to 19.8%), and 17.0% (95% CI: 8.1% to 14.1%), respectively. Overall, the cumulative reinfection rates at 2 and 5 years was 11.0% vs. 18.2% (χ2 = 2.950, *P* = 0.093). The prosthesis-free infection survival rates at 2 and 5 years after DAIR, one-stage revision,1.5-stage revision, and two-stage revision were 85.4% vs. 81.6%, 84.0% vs. 79.2%, 86.2% vs. 82.1%, and 96.0% vs. 83.0%, respectively.

**Conclusion:**

Treatment of a (suspected) TKA-PJI by four strategies—DAIR, one-stage, 1.5-stage, and two-stage revision—had acceptable results based on re-revision and reinfection rates in the long-term (>5 years), resembling the short-term results (<2 years). Focussing on the cultures at the time of initial revision, the incidence of new infections during follow-up was twice that of relapses. Reinfection rates were higher in cases with positive cultures at reimplantation. Patients should be counselled appropriately in this particular situation.

## Introduction

Periprosthetic joint infection (PJI) is one of the most concerning and debilitating complications following joint arthroplasty, significantly impairing patients’ quality of life. According to the literature, the incidence of PJI after primary unilateral total knee arthroplasty (TKA) ranges from 0.7% to 1%, and from 1% to 2% after primary unilateral total hip arthroplasty (Pulido et al., 2008). The rate can be as high as 14% following revision procedures (Garvin et al., 2018), and the risk is even greater in cases of infected revisions, reaching up to 40% (Kunutsor et al., 2016; Bongers et al., 2020). As the number of primary and revision TKA procedures continues to rise, the absolute number of PJI cases is expected to increase (Kurtz et al., 2007). The formation of bacterial biofilms poses a major challenge in the management of PJI (Gbejuade et al., 2015). Treatment typically involves a combination of multiple revision surgeries and prolonged antibiotic therapy (Trampuz and Zimmerli, 2006). For patients who are unable or unwilling to undergo surgical intervention, long-term antibiotic suppressive therapy (AST) may be the only option (Tande and Patel, 2014).

According to the Tsukayama classification, PJI occurring within 4 weeks postoperatively are defined as acute PJI. Most cases of acute PJI can be successfully managed with debridement, antibiotics, and implant retention (DAIR), particularly in patients with stable and well-fixed prostheses, short duration of symptoms, intact soft tissues, and no sinus tract formation. DAIR is considered the optimal treatment for acute PJI, offering advantages such as less invasiveness, reduced pain, and lower cost. In contrast, chronic PJI, which occurs more than 4 weeks after surgery, typically requires implant removal and thorough debridement, combined with systemic antibiotic therapy to eradicate the infection. Currently, the surgical approaches for chronic PJI revision include one-stage revision, 1.5-stage revision, and two-stage revision. Among these, two-stage revision remains the “gold standard” for the treatment of chronic PJI, as recommended by the Infectious Diseases Society of America (IDSA) (Osmon et al., 2013; Chalmers et al., 2018).

Okafor et al. (2023) reported a failure rate of 20% for DAIR and 12.68% for one-stage revision in the treatment of acute periprosthetic joint infection after total knee arthroplasty (TKA-PJI). Siddiqi et al. (2019) found no significant differences between one-stage and two-stage revision for chronic TKA-PJI in terms of reinfection rates (14.0% vs. 24.1%), reoperation rates (19.3% vs. 27.7%), or success rates (78.9% vs. 70.8%). Cai et al. (2021) reported infection recurrence rates of 14.29% for 1.5-stage revision and 9.76% for two-stage revision in chronic TKA-PJI, with no statistically significant difference in infection resolution between the two approaches. Siddiqi et al. (2018) reported a success rate of 79.3% for 1.5-stage revision in chronic TKA-PJI. Belay et al. (2023) found comparable infection control rates between 1.5-stage and two-stage revisions for chronic TKA-PJI (79.3% for both). Bongers et al. (2020) reported reinfection and re-revision rates of 23% and 17%, respectively, following two-stage revision for chronic TKA-PJI. To date, no study has simultaneously evaluated all four surgical strategies for TKA-PJI in terms of postoperative reinfection and re-revision rates.

In this study, we aimed to evaluate: (1) the postoperative reinfection rates following four surgical strategies for TKA-PJI; (2) the microbial profile of recurrent infections; (3) short- and long-term infection-free implant survival rates across the four surgical approaches; (4) re-revision rates due to aseptic loosening and long-term aseptic loosening–free implant survival rates; and (5) the reinfection rate in patients with positive cultures at the time of reimplantation.

## Materials and methods

### Patient selection

This study was approved by the Institutional Review Board and registered at ClinicalTrials.gov under ID: NCT07232771. Data were retrospectively collected from October 2012 to October 2022 for patients with TKA-PJI who underwent treatment with DAIR, one-stage revision, 1.5-stage revision, or two-stage revision at our centre.

Inclusion criteria: (1) Patients who met the diagnostic criteria for TKA-PJI; (2) Patients who underwent one of the following surgical interventions: DAIR, one-stage revision, 1.5-stage revision, or two-stage revision; (3) Patients with complete medical records available; (4) Patients who provided informed consent to participate in the study.

Exclusion criteria: (1) Incomplete medical records; (2) Poor follow-up compliance, including inability to complete scheduled follow-up visits or refusal to participate in follow-up; (3) Follow-up duration less than 2 years.

**Figure 1:**
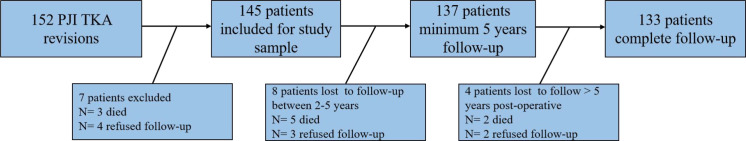
Flow diagram for patient inclusion and exclusion and patients lost to follow-up.

Diagnosis of PJI was established according to the 2018 diagnostic criteria published by the Musculoskeletal Infection Society (MSIS) (Parvizi et al., 2018). Approved by the Institutional Ethics Committee of our hospital, a total of 152 patients with TKA-PJI who underwent surgical treatment were initially identified. After applying inclusion and exclusion criteria, 145 patients were ultimately included in the study: 41 cases underwent DAIR, 25 cases received one-stage revision, 29 cases underwent 1.5-stage revision, and 50 cases underwent two-stage revision. All procedures were performed by 6 experienced surgeons. Patient inclusion, exclusion, and lost to follow-up are summarized in **Figure 1**.

### Study population

Among the 145 patients with TKA-PJI, 65 patients were male and 80 patients were female. The mean age at surgery was 67.47 ± 8.64 years (range: 47–90 years), and the median follow-up was 99 (78.5, 128) months following reimplantation. Patient demographic characteristics, body mass index (BMI), American Society of Anaesthesiologists (ASA) score, and Charlson Comorbidity Index (CCI) are summarized in **Table 1**. The median follow-up after DAIR, one-stage revision, 1.5-stage revision, and two-stage revision were 95 (72,123) months, 103 (81.5,128.5) months, 112 (87.5,137) months, and 100.5 (77.5,135.75) months, respectively.

**Table 1:** Patient demographics, reinfection rates, re-revision rates, reinfection treatment and causes for aseptic re-revision.

Variable	Total (n=145)	DAIR (n=41)	1-stage (n=25)	1.5-stage (n=29)	2-stage (n=50)	p-value
Age (years, X̄ ±S)	67.47 ± 8.64	68.12 ± 9.04	69.92 ± 6.09	66.07 ± 8.13	66.52 ± 9.55	0.309^a^
Gender (n)						0.509^b^
Male	65	18	11	10	26	
Female	80	23	14	19	24	
BMI (kg/m^2^,X̄ ±S)	26.46 ± 1.49	26.77 ± 1.80	26.40 ± 1.24	26.15 ± 1.75	26.42 ± 1.10	0.385^a^
Laterality(n)						0.809^b^
Left	75	20	15	14	26	
Right	70	21	10	15	24	
Total follow-up(month, median, IQR)	99 (78.5, 128)	95 (72, 123)	103 (81.5, 128.5)	112 (87.5, 137)	100.5 (77.5, 135.75)	0.554^d^
ASA classification (n)						0.964^c^
1	26	6	6	5	9	
2	101	29	17	21	34	
3	18	6	2	3	7	
CCI(score)	4.08 ± 0.95	4.05 ± 1.00	4.04 ± 0.89	4.07 ± 0.84	4.12 ± 1.02	0.981^a^
Sinus tract(n,%)	33 (22.8%)	0	6 (24%)	9 (31%)	18 (36%)	<0.001^c^
Duration of the antibiotic treatment (wk, X̄ ±S)	6.96 ± 2.60	6.22 ± 1.93	6.80 ± 1.92	6.90 ± 1.47	7.68 ± 3.57	0.061^a^
MDROs (n,%)	12 (8.3%)	2 (4.9%)	2 (8%)	3 (10.3%)	5 (10%)	0.805^c^
Infection classification (n)						<0.001^c^
Acute infection	57	41	9	7	0	
Chronic infection	88	0	16	22	50	
Reinfection rate (n,%)						0.959^c^
New infection	14 (9.7%)	3 (7.3%)	3 (12%)	3 (10.3%)	5 (10%)	
Relapse	7 (4.8%)	2 (4.9%)	1 (4%)	2 (6.9%)	2 (4%)	
Other	4 (2.8%)	2 (4.9%)	1 (4%)	0	1 (2%)	
Total	25 (17.2%)	7 (17.1%)	5 (20%)	5 (17.2%)	8 (16%)	
Re-revision rate (n,%)						0.803^c^
Septic	17 (11.7%)	4 (9.8%)	5 (20%)	5 (17.2%)	3 (6%)	
Aseptic	8 (5.5%)	2 (4.9%)	1 (4%)	2 (6.9%)	3 (6%)	
Total	25 (17.2%)	6 (14.6%)	6 (24%)	7 (24.1%)	6 (12%)	
Reinfection treatment (n)						0.169^c^
DAIR(S)	5	3	0	0	2	
1.5-stage revision	4	2	2	0	0	
2-stage revision	13	2	3	5	3	
2-stage revision follow by amputation	1	0	0	0	1	
Amputation	1	0	0	0	1	
Arthrodesis	1	0	0	0	1	
Causes of aseptic re-revision (n)						1.000^c^
Tibial and femoral component loosening	2	0	0	1	1	
Femoral component loosening	3	1	0	1	1	
Tibial component loosening	3	1	1	0	1	

^a^ Analysis of variance (ANOVA) with *post hoc* Tukey tests. Quantitative data are expressed as mean ± standard deviation.

^b^ Chi-squared test.

^c^ Fisher’s exact test.

^d^ Kruskal-Wallis H test.

BMI, body mass index; CCI, Charlson comorbidity index; MDROs, multidrug-resistant organisms; DAIR, Debridement, antibiotics and implant retention.

### Diagnosis of infection

The diagnosis of PJI was based on the 2018 Musculoskeletal Infection Society (MSIS) criteria (Parvizi et al., 2018), which require the presence of one of the following: (1) A sinus tract communicating with the prosthesis; (2) Isolation of the same pathogen from two separate tissue or synovial fluid samples obtained from the affected joint; or (3) Meeting at least four out of the following six criteria: (a) Elevated erythrocyte sedimentation rate (ESR) and C-reactive protein (CRP) levels; (b) Elevated synovial fluid white blood cell (WBC) count; (c) Elevated synovial fluid neutrophil percentage (neutrophil differential); (d) Gross intraoperative purulence in the affected joint; (e) Isolation of a microorganism from a single tissue or synovial fluid culture; (f) Histopathological analysis showing more than five neutrophils per high-power field (×400) in five separate fields of periprosthetic tissue.

A total of 145 patients underwent microbial culture either before or during revision surgery. Among them, 44 patients had negative culture results, and 101 had positive cultures. In the DAIR group, 11 cultures were negative and 30 were positive; in the one-stage revision group, 11 were negative and 14 were positive; in the 1.5-stage revision group, 10 were negative and 19 were positive; and in the two-stage revision group, 12 were negative and 38 were positive. The distribution and proportions of isolated microorganisms are summarized in **Table 2**. Polymicrobial infections were identified in 16 patients, all involving two or more distinct microorganisms. Fungal co-infections were detected in 5 patients, and Mycobacterium tuberculosis complex infection was identified in 1 patient. Additionally, multidrug-resistant organisms (MDROs) were isolated in 12 patients.

**Table 2:** Organisms cultured preoperatively or intraoperatively.

Organism	Total (n=145)	DAIR (n=41)	1-stage (n=25)	1.5-stage (n=29)	2-stage (n=50)	p-value
Staphylococcus epidermidis (n,%)	31 (25.4%)	9 (25.0%)	4 (26.7%)	5 (20.0%)	13 (28.3%)	0.896^a^
Staphylococcus aureus (n,%)	29 (23.8%)	9 (25.0%)	2 (13.3%)	6 (24.0%)	12 (26.1%)	0.816^a^
Staphylococcus capitis (n,%)	7 (5.7%)	4 (11.1%)	0	1 (4.0%)	2 (4.3%)	0.538^b^
Escherichia coli	5 (4.1%)	1 (2.8%)	1 (6.7%)	1 (4.0%)	2 (4.3%)	0.921^b^
Streptococcus agalactiae (n,%)	5 (4.1%)	3 (8.3%)	1 (6.7%)	1 (4.0%)	0	0.139^b^
Enterococcus faecalis (n,%)	5 (4.1%)	0	1 (6.7%)	1 (4.0%)	3 (6.5%)	0.367^b^
Staphylococcus haemolyticus (n,%)	4 (3.3%)	0	1 (6.7%)	0	3 (6.5%)	0.248^b^
Candida parapsilosis (n,%)	3 (2.5%)	1 (2.8%)	0	1 (4.0%)	1 (2.2%)	1.000^b^
Klebsiella pneumoniae (n,%)	5 (4.1%)	2 (5.6%)	0	0	1 (2.2%)	0.734^b^
Enterobacter cloacae (n,%)	2 (1.6%)	1 (2.8%)	0	0	1 (2.2%)	1.000^b^
Staphylococcus caprae (n,%)	2 (1.6%)	0	1 (6.7%)	0	1 (2.2%)	0.358^b^
Staphylococcus lugdunensis (n,%)	2 (1.6%)	0	1 (6.7%)	0	1 (2.2%)	0.358^b^
MRSA (n,%)	2 (1.6%)	2 (5.6%)	0	0	0	0.264^b^
Other Organism (n,%)	22 (18.0%)	5 (13.9%)	3 (20.0%)	9 (36.0%)	5 (10.9%)	0.054^a^
Total Organism (n)	122	36	15	25	46	–
No Growth (n)	44	11	11	10	12	–

^a^ Chi-squared test.

^b^ Fisher’s exact test.

MRSA, Methicillin-Resistant Staphylococcus aureus.

### Treatment and surgical technique

Treatment decisions are made based on a comprehensive, multi-dimensional assessment incorporating international consensus, pathogen types, soft tissue status, and radiological findings. Patients with acute PJI who had early postoperative infection (≤4 weeks) or acute hematogenous infection (≤4 weeks), susceptible pathogens without drug-resistant organisms, normal immune function, absence of severe comorbidities, radiologically and intraoperatively confirmed stable prosthesis without loosening or osteolysis, and favourable soft-tissue conditions with no sinus tract formation were indicated for DAIR; one-stage revision was indicated for patients with acute or chronic PJI with susceptible pathogens, no drug-resistant or polymicrobial infection, normal immune function, no sepsis, satisfactory soft-tissue conditions, and definite microbiological identification; one-and-a-half-stage revision was performed in patients with acute or chronic PJI with implant loosening requiring replacement who could not tolerate two-stage revision but did not meet the criteria for one-stage revision (e.g., mild immunosuppression and relatively severe infection); and two-stage revision was indicated for patients with chronic PJI presenting as late postoperative infection (>4 weeks) or chronic infection (>12 weeks), infection caused by drug-resistant bacteria, fungi, polymicrobial pathogens, or culture-negative refractory infection, as well as radiologically and intraoperatively confirmed implant loosening, soft-tissue defects, and sinus tract formation. The detailed methods are as follows.

(1) DAIR revision involves washout, thorough debridement, antibiotics, and all components of implant retention, but polyethylene exchange; (2) One-stage revision involves open debridement of the infected knee, followed by immediate revision by removal and or reimplantation of all components; (3) The 1.5-stage revision involves the use of a functional, articulating antibiotic-loaded knee spacer intended for long-term retention—either indefinitely or until loosening necessitates definitive TKA revision. We standardize the terminology for this procedure as “1.5-stage revision”, as it represents an intermediate strategy between one-stage and two-stage revision for the treatment of PJI following TKA; (4) Two-stage revision, with removal of implants, placement of an antibiotic spacer, and parenteral antibiotic treatment followed by TKA reimplantation.

According to the microbiological results from the preoperative and intraoperative tissue specimens, postoperative antimicrobial treatments were designed by infectious-disease specialists. Since 2015, the usual practice has been to administer postoperative antibiotic therapy intravenously for 2 weeks followed by 4 weeks of oral therapy regardless of the surgical modality. For culture-positive PJI (CP-PJI), targeted therapy was administered using sensitive antibiotics. For culture-negative PJI (CN-PJI), a standardized regimen was adopted: intravenous vancomycin or linezolid plus meropenem for 2 weeks, followed by oral levofloxacin plus rifampin for 4 weeks, combined with vancomycin-loaded bone cement spacers. From 2012 to 2015, total duration of treatment may have been longer; up to 6 months. However, parenteral treatment very rarely exceeded 6 weeks if highly bioavailable oral treatment could be used. The antibiotic-free interval before the 2nd stage operation has been variable, but most often not less than 2 weeks (2–6 weeks). Also, antibiotics have been discontinued after the 2nd stage operation with negative intraoperative cultures and no patient-specific indication for prolonged suppressive antibiotic treatment. In staphylococcal infections, a rifampin-based combination was used when not contraindicated (drug interactions or high risk of adverse reactions) except in two-stage revisions without any foreign material left *in situ*.

### Data collection

Demographic characteristics, treatment strategies, pathogen profiles, rates of implant loosening, reinfection, recurrence, *de novo* infection, culture positivity, infection-free survival, and all-cause revision rates were collected for all enrolled patients. Demographic variables included patient sex, age, BMI, ASA classification, CCI, type of reinfection, and surgical site (laterality). Laboratory data included pathogen identification and microbial culture positivity rates.

### Outcome measures

To determine the reinfection rate, we recorded all adverse events related to subsequent surgical procedures, including reoperations for infection with implant retention and re-revisions with implant exchange. Infections were classified as follows: new infections (growth of a different organism from the initial revision), relapses (growth of the same organism as in the initial revision), or other (no organism identified during the initial revision, but positive cultures obtained at re-revision). To calculate the re-revision rate, we recorded all revision procedures performed for any cause and categorized them as either infection-related or aseptic revisions. Infection-related re-revision was defined as prosthetic removal in patients with positive culture results from joint aspiration and/or intraoperative tissue samples, consistent with clinical or laboratory evidence of infection or presence of a sinus tract. Reinfection and re-revision rates were evaluated at 2-year and 5-year follow-up time points. To address our fifth research question, patients were stratified into three groups based on culture results at the time of reimplantation: those with negative cultures, those with a single positive culture, and those with two or more positive cultures yielding the same pathogen. Reinfection rates were then compared across these groups.

### Statistical analysis

All data were analysed using SPSS software, version 26.0 (IBM Corp., Armonk, New York, USA). Normal distribution quantitative variables are presented as mean ± standard deviation (SD) and were compared using one-way analysis of variance (ANOVA) with Tukey’s *post hoc* test for multiple comparisons. Medians with interquartile ranges for variables with non-Gaussian populations, and multiple comparisons are performed using Kruskal-Wallis H test. Categorical variables are expressed as number of cases (n) and percentage (%). Inter-group comparisons were performed using the chi-square test or Fisher’s exact test, as appropriate. A p-value < 0.05 was considered statistically significant. Kaplan-Meier survival analyses were performed with reinfection (defined as nes infections, recurrence, or other) and aseptic implant loosening (for any reason) as endpoint events, to determine infection-free survival and loosening-free survival rates, respectively.

## Results

### Reinfection rate

**Figure 2:**
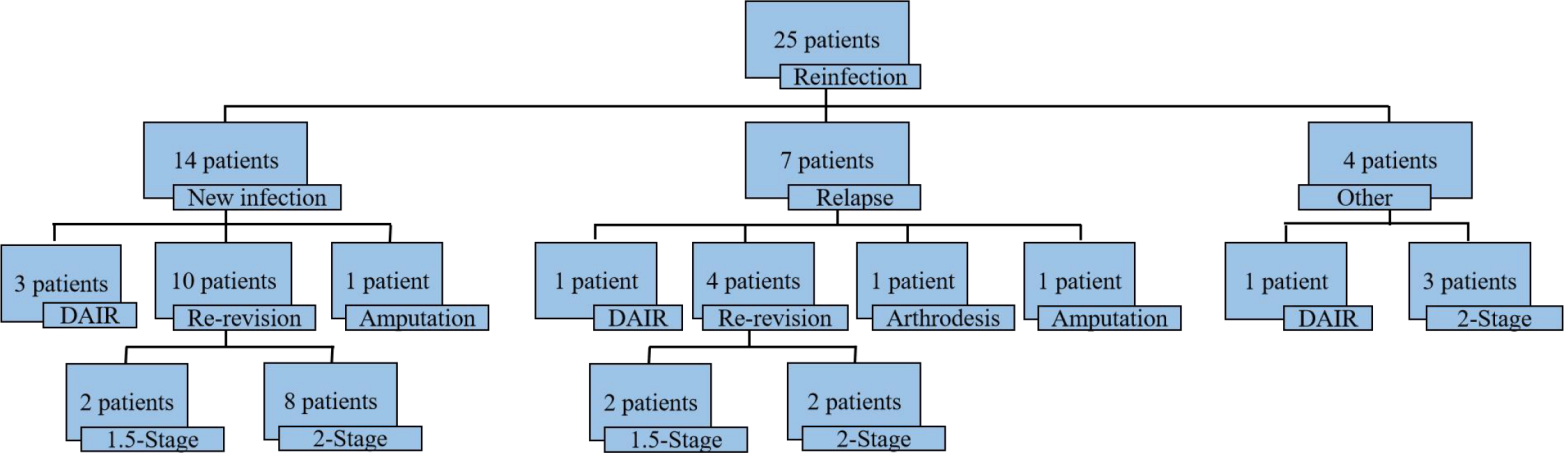
Reinfections, divided by new infections, relapses and other, and how reinfections were treated.

**Figure 3:**
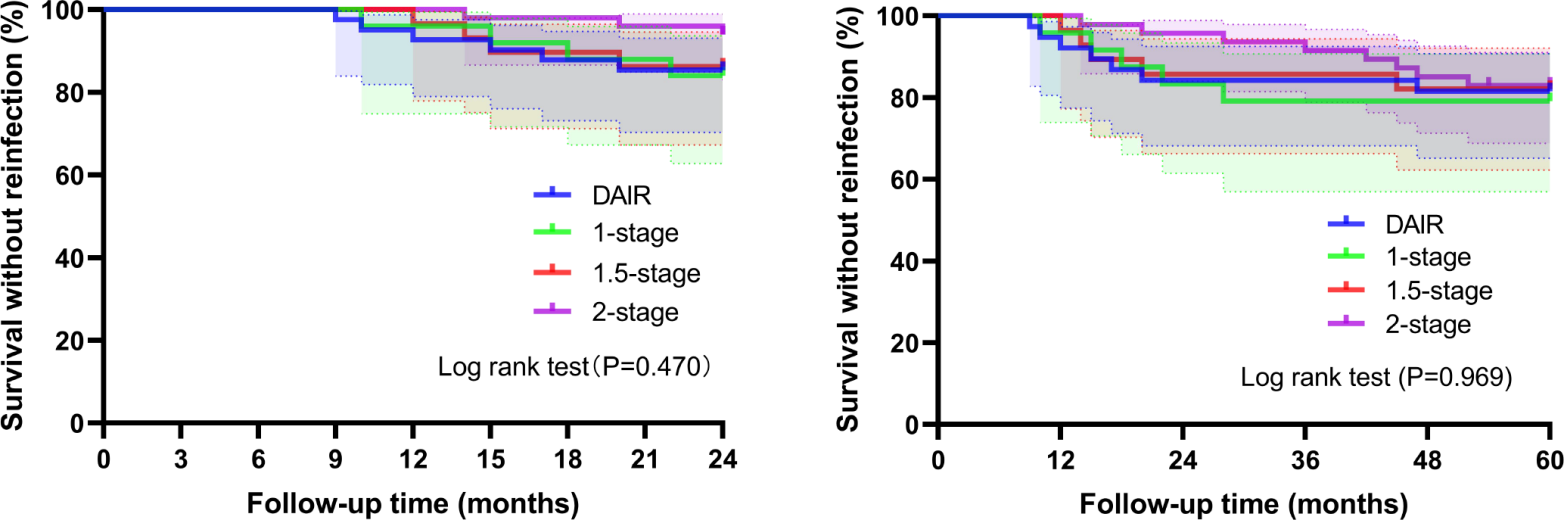
Survival analysis showed no statistically significant difference among the four groups in reinfection at 2 years and 5 years, respectively (Log-rank p=0.470 vs 0.969).

At the most recent follow-up, 25 patients (22%, 25/113) experienced reinfection (**Figure 2**). The mean time to reinfection after reimplantation was 24.5 months (SD14.0), with variation observed across treatment groups (DAIR 18.6, one-stage 18.6, 1.5-stage 21.2, and two-stage 35.5) (**Figure 3**). Of the 25 reinfections, 14 (56%) were new infections, 7 (28%) were relapses, and 4 (16%) were of indeterminate type (**Figure 2**). The mean time to new infection was 17.4 months (SD 16.4), compared to 4.5 months (SD 7.4) for relapses.

The cumulative reinfection rates were as follows: DAIR group: 14.6% (6/41) at 2 years (95% CI: 7.8%–15.1%) and 18.4% (7/38) at 5 years (95% CI: 9.2%–16.4%); One-stage revision group: 16.0% (4/25) at 2 years (95% CI: 9.7%–21.2%) and 20.8% (5/24) at 5 years (95% CI: 11.6%–22.2%); 1.5-stage revision group: 13.8% (4/29) at 2 years (95% CI: 8.4%–18.9%) and 17.9% (5/28) at 5 years (95% CI: 10.0%–19.8%); Two-stage revision group: 4.0% (2/50) at 2 years (95% CI: 3.0%–11.1%) and 17.0% (8/47) at 5 years (95% CI: 8.1%–14.1%) (**Table 3**). Infection-free prosthesis survival rates at 2 and 5 years were: DAIR group: 85.4% vs. 81.6% (χ² = 0.206, p = 0.765); One-stage group: 84.0% vs. 79.2% (χ² = 0.191, p = 0.725); 1.5-stage group: 86.2% vs. 82.1% (χ² = 0.177, p = 0.730); Two-stage group: 96.0% vs. 83.0% (χ² = 4.443, p = 0.047) (**Figure 3**).

**Table 3:** 2-year reinfection rate, 5-year reinfection rate and re-revision rate.

Reinfection rate & re-revision rate	Total	DAIR	1-stage	1.5-stage	2-stage	p-value
2-year reinfection rate, n(%)	16/145 (11.0%)	6/41(14.6%)	4/25 (16.0%)	4/29 (13.8%)	2/50 (4.0%)	0.278^a^
2-year relapses rate, n(%)	5/145 (3.4%)	2/41(4.9%)	1/25 (4.0%)	2/29 (6.9%)	0%	0.254^b^
2-year new infections rate, n(%)	7/145 (4.8%)	2/41(4.9%)	2/25 (8.0%)	2/29 (6.9%)	1/50 (2.0%)	0.557^b^
5-year reinfection rate, n(%)	25/137 (18.2%)	7/38(18.4%)	5/24 (20.8%)	5/28 (17.9%)	8/47 (17.0%)	0.989^a^
5-year relapses rate, n(%)	7/137 (5.1%)	2/38 (5.3%)	1/24 (4.2%)	2/28 (7.1%)	2/47 (4.3%)	0.950^b^
5-year new infections rate, n(%)	14/137 (10.2%)	3/38(7.9%)	3/24 (12.5%)	3/28 (10.7%)	5/47 (10.6%)	0.942^b^
5-year re-revision rate for any reason, n(%)	25/137 (18.2%)	6/38(15.8%)	6/24 (25.0%)	7/28 (25.0%)	6/47 (12.8%)	0.450^a^

^a^ Chi-squared test.

^b^ Fisher’s exact test.

Overall, the cumulative reinfection rates at 2 and 5 years were 11.0% (16/145) and 18.2% (25/137), respectively (χ² = 2.950, p = 0.093). No recurrent infections were observed beyond 5 years postoperatively. Stratified analysis by infection duration (acute vs. chronic) revealed that the cumulative reinfection rate at 2 years was 15.8% vs. 8.0% (χ² = 2.163, p= 0.141), and at 5 years was 20.4% vs. 16.9% (χ² = 0.269,p = 0.604), with no statistically significant differences between groups.

### Re-revision rate

**Figure 4:**
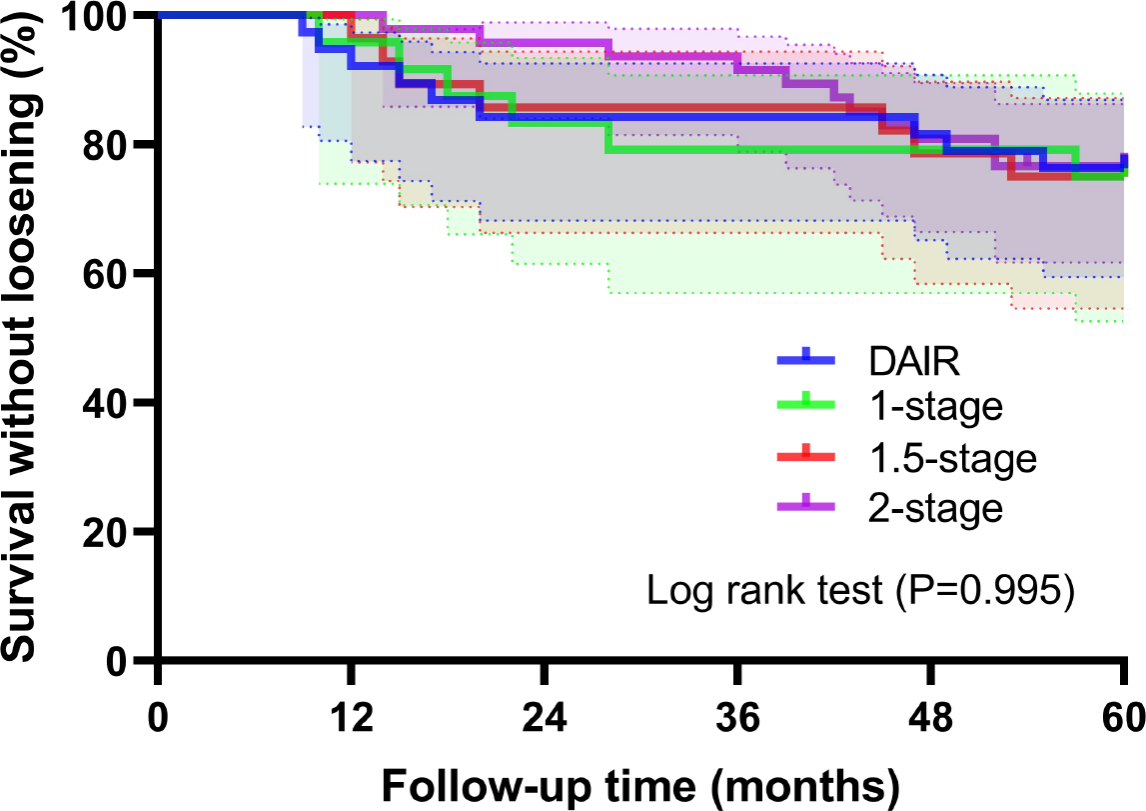
Survival analysis showed no statistically significant difference among the four groups in terms of prosthesis loosening revision for any reason within 5 years (Log-rank p=0.995).

With prosthetic loosening for any reason as the endpoint, the overall 5-year re-revision rate was 18.2% (25/137), comprising 12.4% (17/137) for infection-related loosening and 5.8% (8/137) for aseptic loosening. The 5-year re-revision rates by surgical group were as follows: 15.8% (6/38) in the DAIR group, 25.0% (6/24) in the one-stage revision group, 25.0% (7/28) in the 1.5-stage revision group, and 12.8% (6/47) in the two-stage revision group (**Figure 4**). Stratified analysis by infection duration (acute vs. chronic) also showed that the 5-year revision rate for any cause was 22.2% vs. 15.7% (χ² = 0.944, p = 0.331), and the difference was not statistically significant.

Among the 14 cases of new infections, 3 patients developed acute hematogenous infections at other sites and were treated with one or more DAIR procedures. Ten patients required further revision surgeries (2 cases of 1.5-stage revision and 8 cases of two-stage revision), while one patient was managed conservatively with antibiotics but ultimately underwent amputation due to uncontrolled infection leading to osteomyelitis. Among the 7 cases of relapses, one patient underwent a single DAIR procedure, four patients required additional revision surgeries include 2 cases of 1.5-stage revision and 2 cases of two-stage revision, one patient underwent knee arthrodesis after successful infection control, and one patient underwent amputation. Additionally, in 4 cases, it was unclear whether the infection was new infections or relapses; one patient underwent DAIR, and three patients underwent two-stage revision.

Seventeen patients (12.4%) underwent re-revision surgery due to infection-related prosthetic loosening. The mean interval between the initial revision and re-revision for infection-related loosening was 22.6 months (SD 12.8). Eight patients (5.8%) underwent re-revision surgery due to aseptic prosthetic loosening, with a mean interval of 49.4 months (SD 6.1) between the initial revision and re-revision for aseptic loosening. Among the re-revision surgeries for aseptic loosening, there were 2 cases involving bilateral component revisions, 3 cases involving femoral component revisions, and 3 cases involving tibial component revisions.

### Positive cultures at reimplantation

**Figure 5:**
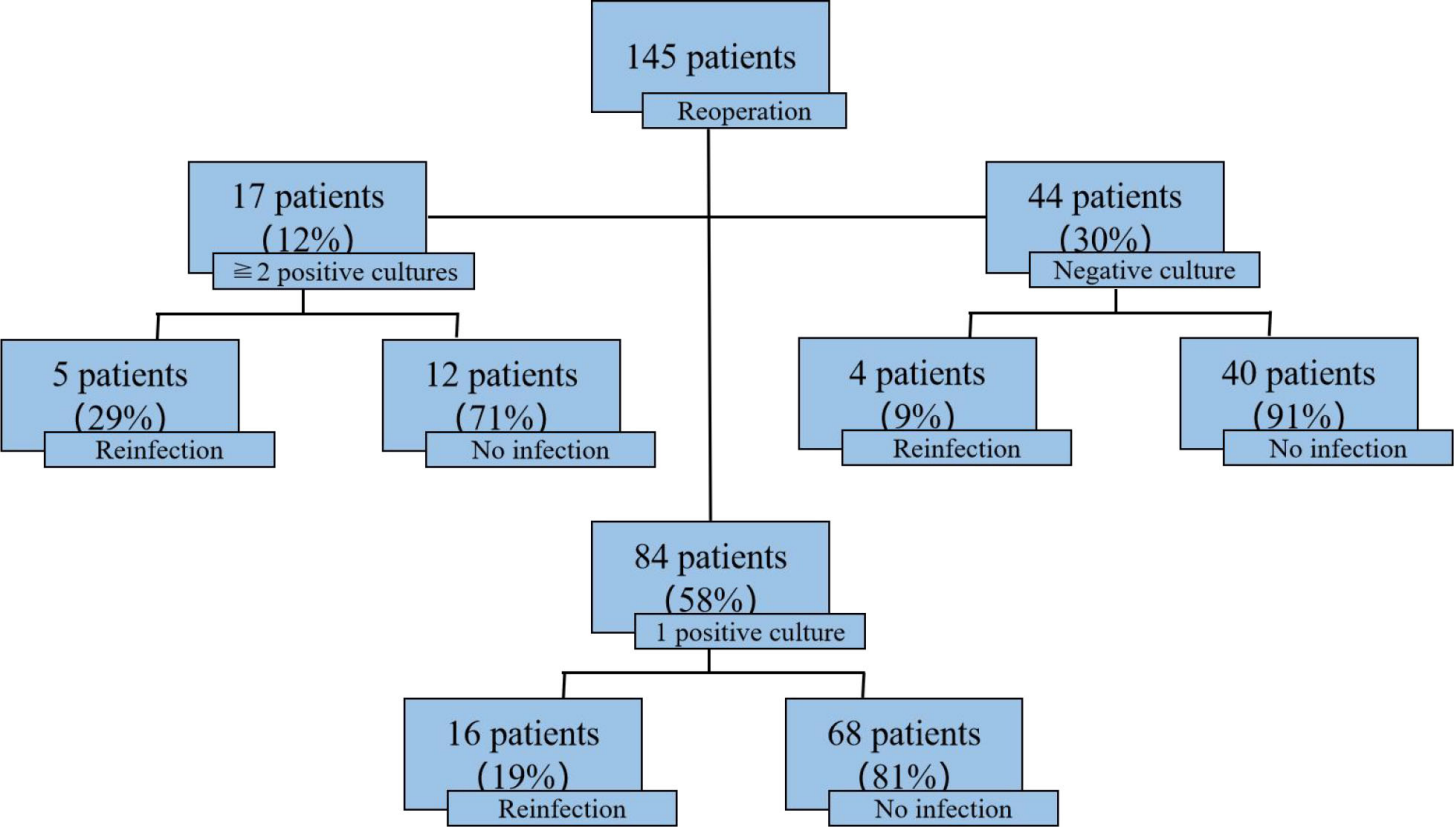
Reinfection rates divided by culture results at reoperation.

There was no statistically significant difference in reinfection rates among the three groups (χ² = 4.00, p = 0.135): 29% (5/17) in patients with two or more positive cultures at reimplantation, 19% (16/84) in those with one positive culture, and 9% (4/44) in those with negative cultures (**Figure 5**). Overall, patients with positive cultures at the time of reimplantation had higher reinfection rates. Compared to patients with fewer than two positive cultures, those with two or more positive cultures tended to have higher reinfection rates, although the difference was not significant (29% vs. 19%, χ² = 0.922, p = 0.337). Similarly, patients with one positive tissue culture had a higher reinfection rate than those with no positive cultures, but this difference also did not reach statistical significance (19% vs. 9%, χ² = 2.171, p = 0.141). However, patients with two or more positive cultures had a significantly higher reinfection rate compared to those with no positive cultures (29% vs. 9%, χ² = 4.026, p = 0.045).

Among the 25 patients who experienced reinfection, 7 were in the DAIR group, 5 in the one-stage revision group, 5 in the 1.5-stage revision group, and 8 in the two-stage revision group. Microbiological analysis revealed a new organism (new infection) in 14 patients, the same organism as the initial infection (relapse) in 7 patients, and an indeterminate infection type in 4 patients (microbial isolates are listed in **Tables 4**, **5**).

**Table 4:** Organisms cultured at reinfection or reimplantation.

Organism	Total (n=25)	DAIR (n=7)	1-stage (n=5)	1.5-stage (n=5)	2-stage (n=8)	p-value#
Staphylococcus epidermidisn(n,%)	4 (14.3%)	1 (12.5%)	1 (20%)	1 (16.7%)	1 (11.1%)	1.000
Staphylococcus capitis(n,%)	4 (14.3%)	2 (25%)	0	1 (16.7%)	1 (11.1%)	0.810
Staphylococcus aureus(n,%)	4 (14.3%)	0	2 (40%)	1 (16.7%)	1 (11.1%)	0.278
Escherichia coli(n,%)	3 (10.7%)	0	1 (20%)	2 (33.3%)	0	0.067
Staphylococcus haemolyticus(n,%)	3 (10.7%)	0	0	0	3 (33.3%)	0.118
Enterococcus faecalis(n,%)	2 (7.1%)	1 (12.5%)	1 (20%)	0	0	0.421
Streptococcus agalactiae(n,%)	2 (7.1%)	1 (12.5%)	0	1 (16.7%)	0	0.667
MRSA(n,%)	2 (7.1%)	1 (12.5%)	0	0	1 (11.1%)	1.000
Cutibacterium acnes(n,%)	2 (7.1%)	1 (12.5%)	0	0	1 (11.1%)	1.000
Enterobacter cloacae(n,%)	1 (3.6%)	1 (12.5%)	0	0	0	0.679
Staphylococcus lugdunensis(n,%)	1 (3.6%)	0	0	0	1 (11.1%)	1.000
Total organism(n)	28	8	5	6	9	–
No Growth(n)	0	0	0	0	0	–

#Fisher’s exact test.

MRSA, Methicillin-Resistant Staphylococcus aureus.

**Table 5:** Organisms of new infections and relapses.

DAIR (n=5)		1-stage (n=4)		1.5-stage (n=5)		2-stage (n=7)	
New organism	Same organism	New organism	Same organism	New organism	Same organism	New organism	Same organism
Staphylococcus capitis (2)	Staphylococcus epidermidis	Staphylococcus aureus	Staphylococcus epidermidis	Staphylococcus epidermidis	Staphylococcus capitis	Staphylococcus capitis	Staphylococcus epidermidis
Cutibacterium acnes	Streptococcus agalactiae	Escherichia coli	–	Staphylococcus aureus	Escherichia coli	Staphylococcus lugdunensis	Staphylococcus aureus
Enterobacter cloacae	–	Enterococcus faecalis	–	Streptococcus agalactiae	–	MRSA	Staphylococcus haemolyticus
–	–	–	–	Escherichia coli	–	Staphylococcus haemolyticus(2)	–

MRSA, Methicillin-Resistant Staphylococcus aureus.

## Discussion

This retrospective study assessed four surgical strategies (DAIR, one-stage, 1.5-stage, two-stage revision) for TKA-PJI, addressing the critical gap of simultaneous comparison across all approaches by analysing reinfection rates, microbial profiles, implant survival, and intraoperative culture impacts.

For reinfection and infection-free survival, no universally superior strategy emerged, but two-stage revision offered early advantages (Bongers et al., 2020; Cai et al., 2021; Belay et al., 2023): at 2 years, its 4.0% reinfection rate was lower than DAIR (14.6%), one-stage (16.0%), and 1.5-stage (13.8%) groups (p=0.278). Notably, the two-stage group was the only one with a statistically significant decline in 5-year vs. 2-year infection-free survival (83.0% vs. 96.0%, p=0.047), indicating late reinfections (2–5 years postoperatively) erode early benefits. This aligns with Siddiqi et al.’s (Siddiqi et al., 2019) finding of no significant difference in reinfection rates between one-stage and two-stage revision (14.0% vs. 24.1%) and extends this conclusion to DAIR and 1.5-stage revision. By 5 years, reinfection rates were nearly identical across groups (17.0%–20.8%, p=0.989), with no recurrences beyond 5 years—identifying this as the “high-risk period” to guide follow-up. DAIR’s 5-year reinfection rate (18.4%) was slightly lower than prior reports (20%) (Okafor et al., 2023), attributed to strict patient selection. This suggests that DAIR remains a viable option for acute PJI, particularly in patients who prioritize minimally invasive care, but requires careful patient selection to balance its advantages (lower invasiveness, reduced cost) against its long-term reinfection risk (Urish et al., 2018; Choo et al., 2019; Zhang et al., 2020; Alamino et al., 2025; Zellner et al., 2025).

In terms of microbial profiles of TKA-PJI, staphylococcal species are the dominant pathogens, while MDROs and polymicrobial infections still pose considerable challenges to the effective management of this condition. The microbial profile of both initial and recurrent TKA-PJI in our cohort underscores the central role of staphylococcal species—consistent with global PJI epidemiology (Parvizi et al., 2018). Staphylococcus epidermidis (25.4%) and S. aureus (23.8%) were top initial pathogens, while recurrent infections were driven by S. epidermidis, S. capitis, and S. aureus (14.3% each). Furthermore, subgroup analysis stratified by pathogen type demonstrated that S. aureus and coagulase-negative staphylococci (CoNS) accounted for the highest proportion among all TKA-PJI cases. This dominance of CoNS highlights the challenge of biofilm formation (Gbejuade et al., 2015), as CoNS are notorious for adhering to prosthetic surfaces and evading antibiotic therapy—even with prolonged systemic or local administration. Notably, 16 patients (11.0%) had polymicrobial infections, and 12 (8.3%) had MDROs. Polymicrobial infections are often associated with worse outcomes due to synergistic bacterial interactions and broader antibiotic resistance (Marculescu and Cantey, 2008; Wimmer et al., 2016; Auñón et al., 2025), while MDROs limit treatment options (e.g., requiring vancomycin or linezolid for MRSA). In addition, MDROs infections were mainly observed in cases with failed DAIR and late chronic infection, carrying a higher risk of reinfection than non-MDROs infections. In recurrent infections, we observed a shift toward “atypical” pathogens: for example, Staphylococcus haemolyticus (a CoNS) was isolated in 3 of 8 recurrent infections in the two-stage group, and Cutibacterium acnes (a slow-growing anaerobe) was identified in 2 cases. Recurrent infections were also predominantly caused by staphylococcal species, which were highly consistent with the initial causative pathogens. This suggests that late reinfections may stem from persistent, low-virulence pathogens that evade initial debridement or antibiotic therapy, and biofilm-mediated persistent infection also serves as a key mechanism underlying recurrence—emphasizing the need for prolonged culture incubation (e.g.,14 days for anaerobes) and comprehensive microbial testing (e.g., 16S rRNA sequencing) to avoid missed diagnoses. Moreover, Gram-negative bacteria, fungi, and polymicrobial infections were more commonly seen in patients with more complex conditions and poor soft tissue conditions, who were more likely to undergo one-and-a-half-stage or two-stage revision.

When examining re-revision rates across the four strategies for TKA-PJI, aseptic loosening accounts for a relatively small proportion of re-revision causes compared to infection-related loosening, yet it remains a consistent concern that requires clinical attention during long-term follow-up. The 5-year overall re-revision rate was 18.2%, with infection-related loosening (12.4%) predominating over aseptic loosening (5.8%). Aseptic loosening rates were similar across groups (4.0%–6.9%, p=0.97). This is consistent with prior studies (Cai et al., 2021; Belay et al., 2023), which report low rates of aseptic loosening in PJI revisions, likely due to improvements in prosthetic fixation (e.g., cemented implants with antibiotic-loaded cement) and soft tissue preservation. Notably, the one-stage and 1.5-stage groups had the highest 5-year re-revision rates (25.0% each), driven by both infection-related and aseptic causes. For 1.5-stage revision, our findings support Belay et al.’s (Belay et al., 2023) observation of comparable infection control to two-stage revision (79.3% vs. 79.3%) but highlight that its use of a long-term antibiotic spacer may increase late loosening risk(e.g., spacer wear or bone loss), necessitating re-revision. This suggests that 1.5-stage revision is best suited for patients intolerant of two-stage revision’s prolonged antibiotics but needing more robust control than DAIR (Cai et al., 2021; Festa et al., 2025).

In the context of TKA-PJI revision surgery, intraoperative culture positivity—especially when multiple cultures yield positive results—serves as a valuable predictor of subsequent reinfection, highlighting its clinical significance in assessing post-revision infection risk. A novel finding: intraoperative culture positivity (especially ≥2 positive cultures) predicted reinfection. Patients with ≥2 positive cultures had a significantly higher 5-year reinfection rate (29% vs. 9%, p=0.045) than those with negative cultures, while single positive cultures showed no statistical difference (19% vs. 9%, p=0.141). Our findings are consistent with those of Şenel A et al (Şenel et al., 2023). who reported a lower reinfection rate in two-stage revision studies with a higher proportion of culture-negative cases. Also, this aligns with the MSIS 2018 criteria (Parvizi et al., 2018), which prioritize multiple positive cultures for PJI diagnosis, and extends this to prognosis: multiple positive cultures may indicate persistent biofilm or occult infection, even after debridement. Clinically, this suggests that patients with two or more positive cultures at reimplantation may benefit from extended AST or closer monitoring, even if they meet criteria for “successful” revision. For example, AST could be considered for 3–6 months post-reimplantation in this high-risk subgroup to reduce reinfection risk—a strategy that has shown promise in patients unable to undergo surgery (Tande and Patel, 2014).

This study has several limitations that should be considered when interpreting its findings. First, its retrospective design introduces inherent biases, such as selection bias (e.g., surgeons may have preferred two-stage revision for more severe infections, though baseline characteristics (age, BMI, ASA score, CCI) were balanced across groups (p>0.05). Second, while the mean follow-up of 8.7 years is longer than most TKA-PJI studies, the small sample size (n=145, with subgroups as small as n=25 for one-stage revision) limits statistical power—particularly for detecting small but clinically meaningful differences in reinfection rates. Third, we did not collect data on antibiotic regimens (e.g., type, duration, route of administration), which may have influenced reinfection outcomes. Finally, we did not assess patient-reported outcomes (PROs) (e.g., pain, function), which are critical for evaluating the clinical relevance of reinfection. Future studies should address these limitations with prospective, multicentre designs that: (1) standardize antibiotic regimens to isolate the effect of surgical strategy; (2) include PROs to link reinfection to patient-centric outcomes; (3) evaluate the role of AST in patients with multiple positive cultures at reimplantation.

## Conclusion

In conclusion, our long-term data demonstrate that all four surgical strategies for TKA-PJI—DAIR, one-stage, 1.5-stage, and two-stage revision—achieve comparable 5-year reinfection rates, despite differences in early infection control. The choice of strategy should therefore be guided by patient-specific factors: (1) DAIR for acute PJI with stable prostheses and no sinus tracts; (2) one-stage revision for patients who cannot tolerate prolonged antibiotic courses or delayed reimplantation; (3) 1.5-stage revision as an intermediate option for patients requiring long-term spacer retention; and (4) two-stage revision for chronic PJI or cases with extensive bone loss, where early infection control is prioritized. By tailoring treatment to individual patient needs and monitoring for 5 years postoperatively, clinicians can optimize outcomes for TKA-PJI.

## Data Availability

The original contributions presented in the study are included in the article/**Supplementary Material**. Further inquiries can be directed to the corresponding authors.
